# Face Mask and Tear Film Stability: A Pilot Study of the Objective Measurement of Tear Break-Up Time

**DOI:** 10.3390/jcm12247727

**Published:** 2023-12-16

**Authors:** Karim Mohamed-Noriega, David E. Charles-Cantu, Jibran Mohamed-Noriega, Braulio H. Velasco-Sepúlveda, Fernando Morales-Wong, Gerardo Villarreal-Méndez, Jesús Mohamed-Hamsho

**Affiliations:** Department of Ophthalmology, University Hospital and Faculty of Medicine, Autonomous University of Nuevo León (UANL), Avenida Francisco I Madero 3501 y Avenida José Eleuterio González (Gonzalitos) S/N, Colonia Mitras Centro, Monterrey 64460, Nuevo León, Mexico; david.charlescnt@uanl.edu.mx (D.E.C.-C.); jibran.mohamednrg@uanl.edu.mx (J.M.-N.); braulio.velascosplv@uanl.edu.mx (B.H.V.-S.); fernando.moraleswn@uanl.edu.mx (F.M.-W.); gerardo.villarrealmnd@uanl.edu.mx (G.V.-M.); jesus.mohamedhms@uanl.edu.mx (J.M.-H.)

**Keywords:** tear break-up time, face mask, dry eye disease, new normality, MADE

## Abstract

(1) Background: Mask-associated dry eye (MADE) has been associated with increased dry eye symptoms, apparently due to reduced tear break-up time (TBUT). This study aimed to determine the short-term impact of surgical face mask (FM) on tear film stability by measuring non-invasive tear break-up time (NIBUT). (2) Methods: Twenty-six healthy participants had NIBUT evaluated without FM, with surgical FM and with a surgical FM secured to the skin with adhesive tape (TFM). NIBUT-first was measured with Keratograph 5M (K5M, Oculus, Wetzlar, Germany). Each participant had NIBUT measured in four sessions on four consecutive days. Session 1: without FM vs. with FM. Session 2: with FM vs. without FM. Session 3: without FM vs. with TFM. Session 4: with TFM vs. without FM (3). The time between each measured setting was 2 min. Results: The mean ± SD NIBUT without FM was 8.9 ± 3.7, with FM 10.2 ± 4.1, and with TFM 8.4 ± 3.8 s. No significant differences were observed in NIBUT in any of the evaluated settings: without FM vs. with FM (*p* = 0.247), without FM vs. with TFM (*p* = 0.915), and with FM vs. with TFM (*p* = 0.11). (4) Conclusions: This study did not find a significant short-term effect of FM on NIBUT. Other variables or longer periods of exposure might trigger the symptoms and ocular surface alterations in MADE.

## 1. Introduction

When the COVID-19 pandemic began, organizations like the World Health Organization (WHO) and the Center for Disease Control and Prevention (CDC) recommended the widespread use of face masks to reduce the spread of the disease [1,2]. Since then, face masks have become part of the new normality in society worldwide, and an association between their use and increased incidence of dry eye symptoms has been made in what some have referred to as mask-associated dry eye (MADE) [3,4,5]. The evidence in this regard includes anecdotal comments [3], prospective studies that use questionaries that evaluate only symptoms [4,6,7] and prospective studies that evaluate both symptoms and signs [8,9,10,11]; some of them are descriptive case series, and some others are comparative studies.

The definition of DED according to the TFOS DEWS II is “Dry eye is a multifactorial disease of the ocular surface characterized by a loss of homeostasis of the tear film, and accompanied by ocular symptoms, in which tear film instability and hyperosmolarity, ocular surface inflammation and damage, and neurosensory abnormalities play etiological roles” [12]. It is hypothesized that a face mask (or its incorrect fitting) causes eye irritation and dry eye disease (DED) by directing air leakage upwards over to the ocular surface, producing evaporation of the tear film and subsequent reduction in tear break-up time (TBUT) [3,5,6,13,14]. This would be compatible with evaporative DED, where the tear film is unstable and tear break-up time is reduced [12,15,16].

Various types of face masks have emerged, and these can be broadly categorized into three primary types: N95 respirators, surgical masks, and cloth masks. The N95 respirators are constructed from polypropylene fibers; they offer a high level of filtration efficiency by capturing particles as small as 0.3 microns. The surgical masks are typically composed of multiple layers of non-woven materials and offer comparatively lower filtration efficacy than the N95 respirators. The cloth masks are made from diverse materials such as cotton or polyester blends, are reusable and serve as a sustainable option for everyday use but have an even lower filtration efficacy than surgical and N95 face masks. The impact of different face mask designs on the induction of MADE symptoms is not well studied, but it has been described that different face mask designs can have different impacts on the induction of MADE symptoms [10,17]. One study found that up to 45% of the general population can present improper face mask wear mainly due to air leakage, mostly to gaps at the upper face mask edge [18]. Some studies have shown that interventions that cause increased airflow over the eye, such as continuous positive airway pressure (CPAP), negatively affect the ocular surface by decreasing TBUT and producing symptoms of DED [19,20]. This airflow damage mechanism may be similar to that generated by the exhaust airflow that passes through the top of the face mask when it is not properly sealed. Face mask airtightness is frequently suboptimal even in health care professionals, mainly is due to gaps in the upper face mask border as evidenced by air flowing out [21,22]. To avoid this air leakage, it is a common clinical practice to secure the upper margin of the face mask to the skin using adhesive tape [23,24].

Tear film stability is essential to a healthy ocular surface. TBUT is an important component to all dry eye evaluations, which is conventionally measured by instilling fluoresceine (FBUT), but it can also be measured objectively and non-invasively [25,26]. Non-invasive break-up time (NIBUT) was defined by Mengher in 1985 as “the time from the last full flicker to the first distortion of the rings (of Placido) projected in the tear film” [27]. It is a non-invasive measurement when it “does not involve fluorescein instillation, flicker is natural, unforced or suppressed and there is no contact between the measuring instrument and the eye or eyelids” [28]. Multiple automated systems measure NIBUT, such as Keratograph 5M (K5M, Oculus, Wetzlar, Germany). The K5M obtains the time in seconds (s) of the first tear break-up time (NIBUT-first) and the average tear break-up time (NIBUT-average), which is the mean of all tear film break-ups happening over the entire corneal surface before blinking [29]. There are inherent differences and similarities between NIBUT and FBUT which are controversial [30,31]. Some studies have found that NIBUT values are higher than NIBUT; on the contrary, other studies report that FBUT has greater break-up times than NIBUT [32]. Regarding their correlation and interchangeability, NIBUT has been correlated with FBUT in patients with dry eye, but not correlation was reported on healthy controls without dry eye [33]. The main advantage of NIBUT is that it does not depend on the operator, does not require fluoresceine installation, and as previously mentioned, generates a first and average value. The disadvantages are that it depends on expensive equipment, and the values cannot be interchanged between equipment. The advantage of FBUT is that is widespread in clinical practice and does not need specialized expensive equipment. A disadvantage is its limited reliability and repeatability that depends on the evaluation, technique, time of measurement after fluoresceine instillation, fluoresceine concentration, and volume. Invasive assessments like FBUT are even less repeatable than objective measurements like NIBUT, and some studies have found better repeatability with NIBUT in successive measurements than with FBUT [34].

It is important to understand the physiological mechanisms that contribute to DED symptoms associated with face masks, since their use will surely continue for the foreseeable future, especially in health-care settings. Tear film stability assessment is an essential evaluation to perform on patients with DED. Before the COVID pandemic, the NIBUT was evaluated without a face mask. Often during the pandemic and even currently daily, the NIBUT and FBUT are still being performed with the face mask on. When evaluating a symptomatic patient with MADE, we may want to evaluate the change in NIBUT with and without a mask. Furthermore, if NIBUT is shown to be affected, this might interfere and compromise the reliability of tear film stability tests when assessing DED in the clinic with the patient wearing a face mask. This study aims to determine the effect of surgical face mask use on NIBUT as measured with K5M. It provides information on the short-term impact of face mask on NIBUT in a clinical evaluation.

## 2. Materials and Methods

This was a prospective, experimental, comparative, and transversal study. Staff from the ophthalmology department at the University Hospital “Dr. José E. González” of the autonomous university of Nuevo León (UANL) were invited to participate and they were consecutively included. The study was approved by the institutional ethics committee and was performed in accordance with good clinical practices and with the declaration of Helsinki. Participants were properly informed of the study and its procedures and were given appropriate consent forms to read. Written informed consent was obtained. Inclusion criteria were older than 18 years of age, both genders, no history of eye diseases except dry eye, no history of eye surgery except LASIK more than one year ago and no use of eye drops except artificial tears. In order to limit bias from the possible effect of lubricating eye drops, no artificial tears were applied for at least 2 h before study measurements [35]. Participants with systemic diseases such as diabetes, hypothyroidism or respiratory tract disease were excluded. Those with red eye, discharge, photosensitivity, epiphora, symptoms compatible with adenoviral or allergic conjunctivitis, recent eye surgery, use of eye drops other than artificial tears and dry eye requiring application of artificial tears more than four times daily were also excluded.

**Figure 1:**
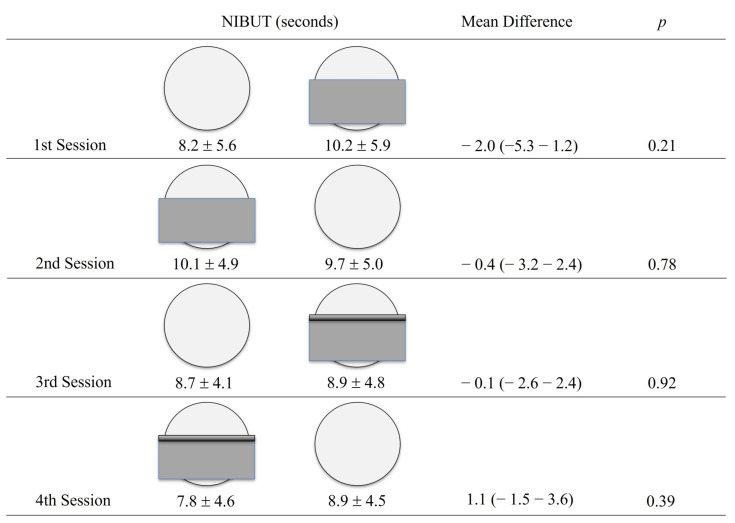
Description of each measuring session and the mean ± standard deviation non-invasive break-up time (NIBUT) values. The mean difference refers to the difference between the two face mask settings evaluated in each session and is expressed as a mean (min–max). The 1st session compared NIBUT without a face mask versus with a face mask. The 2nd session compared NIBUT with a face mask versus without a face mask. The 3rd session compared NIBUT without a face mask versus with a tape-secured face mask. The 4th session compared NIBUT with a tape-secured face mask versus without a face mask. No significant differences in mean NIBUT values were observed in any of the measuring sessions.

Tear film stability was measured with the NIBUT-first test using the Keratograph K5M. with the TF-SCAN software (V 6.11r71) according to the manufacturer’s specifications. The NIBUT-first was chosen over the NIBUT-average because the former resembles clinical TBUT. It was decided to use the NIBUT-first because it measures the first break on the ear film detected by the keratography, it resembles more FBUT measures that also records the first break on the tear film. The NIBUT-first have previously been used to measure tear-films stability in published studies about MADE [7,11,36,37], and in important clinical trials and cohort studies about DED such as the DREAM study [38]. Average measures the average number of seconds in which all tear film ruptures occur before blinking and therefore does not measure the time in which the first tear film rupture occurs. Routinely in our eye center, the area where the Keratograph K5M. is located and where the test is performed has central air conditioning and no windows. This ensures the temperature is constantly 24° Celsius all day in all tests. In addition, it is ensured that the air vents from the climate do not blow directly towards the Keratograph K5M. To evaluate the short-term impact on NIBUT that face mask might have we measured the NIBUTin three different settings: without a face mask, with a face mask (Prophytech disposable three-layered surgical face mask by Zeyco), and with a face mask secured to the skin with adhesive tape. The adhesive tape was used in such a way that it covered the entire top part of the face mask. All participants had their NIBUT measured in four sessions on four consecutive days. Each session consisted in measuring NIBUT with two of the three different settings. In the 1st session, NIBUT was measured first without a face mask and then with a face mask. In the 2nd session, the order was reversed and NIBUT was measured first with a face mask and then without a face mask. The 3rd and 4th sessions followed the same order as the 1st and 2nd sessions, but the face mask was secured to the skin with adhesive tape (Figure 1). The NIBUT of the first and second evaluated settings were taken consecutively one after the other waiting a 2 min for the participant to put or remove the face mask. The actual NIBUT value for each setting was the average of the first 3 good-quality measurements achieved. NIBUT tests of poor quality were eliminated, such as those when the patient blinked at the time of capturing the NIBUT or the patient moved, and the NIBUT image was blurry or incomplete or no value was recorded. All the participants were evaluated only the right eye, between 11:00 h and 15:00 h, by the same technician, with the same equipment and in the same room and lighting conditions.

A sample size calculation was used for the comparison of two means in the same population using the G*Power software (Version 3.1.9.7) [39]. With an expected 25% reduction in NIBUT with face mask and a bilateral significance of 0.05 given a power of 80%, at least 23 study subjects were required. The difference in means of first NBUT in healthy and dry eye disease individuals from a previous study that used Keratograph K5M were used as a reference where they found a 25% difference in NIBUT-first, being larger in healthy participants [34]. Descriptive statistics are presented as mean ± standard deviation (SD). Inference statistics are presented as 95% confidence intervals (CI). All data sets were tested for normality using the Shapiro–Wilk test. Since data were normally distributed, an independent Student’s *t*-test was used. Statistical significance was considered when *p* ≤ 0.05. Bland–Altman analysis was performed to evaluate agreement between measurements. Data were analyzed using the SPSS software (IBM Corp. Released 2012. IBM SPSS Statistics for Windows, Version 21.0. Armonk, NY, USA: IBM Corp.).

## 3. Results

A total of 26 participants were included in the study. The mean ± SD age was 30.3 ± 5.9 years (Range: 23–43). 15 (57.6%) participants were female. The mean ± SD NIBUT for all measurements without a face mask was 8.9 ± 3.7 s, with a face mask was 10.2 ± 4.1, and with a tape-secured face mask was 8.4 ± 3.8. There were no statistically significant differences in NIBUT values between the measurements without a face mask, with a face mask or with a tape-secured face mask (*p* > 0.05) as shown in Table 1. The mean NIBUT value for each of the measuring sessions is presented in Figure 1 and no significant differences in NIBUT were observed in any of the four sessions.

To assess whether the day of the session influenced the NIBUT value, the NIBUT without a face mask from each of the four sessions were compared as follow. By grouping the measurements without a face mask of the first and second sessions and comparing them against the measurements without a face mask of the third and fourth sessions, it was found that the day of measurement had no statistically significant effect on NIBUT values (*p* = 0.91). This grouping was used to neutralize the possible effect that the order in which the measurements are made in each session may have. Similarly, to assess whether the order in which NIBUT was measured in each session influenced the NIBUT value, the NIBUT without a face mask from each of the four sessions were compared as follow. By grouping the measurements without a face mask of the first and third sessions and comparing them against the measurements without a face mask of the second and fourth sessions, it was found that the order of measurement made no statistically significant effect on NIBUT values (*p* = 0.47).

The intraclass correlation coefficient (ICC) for the sessions without face mask was moderate with a value of 0.51, the sessions with face mask showed a poor ICC of 0.11, and the sessions with tape-secured facemask showed a poor ICC of 0.31. The Bland–Altman plot analysis of agreement between NIBUT measurements without face mask showed that the mean difference between measurements was small but with wide limits of agreement (Supplementary Figure S1). Scatter-Plot analysis of each of the four sessions found a moderate to weak positive agreement between NIBUT values with and without face mask, and several outliers were found. However, no effect of face mask use on NIBUT values was observed (Supplementary Figure S2). The results of both agreement analyses imply that while the instrument of measurement may have high inherent variability, the NIBUT itself was also highly variable among and within participants.

## 4. Discussion

The present study objectively evaluated the short-term effect of face mask use on tear film stability and did not find significant changes in NIBUT as measured with K5M. The findings of the current study do not fully support the hypothesis that MADE symptoms are explained by an immediate reduction in NIBUT after removal of the face mask. It is known that symptoms of DED often do not correlate well with all objective signs and tests because of the multifactorial nature of the disease [40,41]. If the increase in DED symptoms related with face mask use is not associated with an immediate reduction in NIBUT, perhaps the extended time of face mask use or another property in the intricated and multifactorial DED pathophysiology might be responsible [15].

The association of DED symptoms with face mask use has been previously described in what is now known as MADE [3,7,13]. A recent online survey found an increase in self-reported DED symptoms while wearing a face mask in 18.3% of the participants [4]. A study on DED patients compared their Ocular Surface Disease Index (OSDI) scores before the COVID pandemic and during the COVID pandemic and found a significant increase in OSDI score only in heavy face mask users (≥6 h/day, and ≥5 days/week, in the last 2 months) when compared with regular face mask users [42]. In addition, several studies have reported that female patients and those with previous DED had a greater increase or worsening of dry eye symptoms with face mask use [4,7,42]. Another survey found that participants using face mask 3–6 h/day had higher OSDI scores, approximately half showed worsening of DED symptoms while wearing the face mask, and those with prior DED had greater worsening of OSDI while wearing the face mask [7]. However, all these studies only evaluated dry eye symptoms, but did not evaluate signs and other clinical evaluations of the ocular surface. 

Several studies have evaluated the effect of prolonged face mask on TBUT. A recent study on DED patients and healthy controls found that after 3 months of face mask use a significant reduction in FBUT was observed only in those using face mask > 6 h daily, and a significant increase in DED symptoms was observed only in DED patients [9]. Another study evaluated symptoms and signs of DED after 8 h of continuous face mask use and found a significant increase in dry eye symptoms and a reduction in FBUT [8]. A similar study evaluated changes in NIBUT after 9 h of continuous use of face mask while working and also found a significant reduction in NIBUT at the end of the day [43]. However, a control group was not evaluated, and it is well known that dry eye symptoms worsen during the day, being more at the end of the day. Another study on patients with DED using a face mask found a significant improvement in NIBUT after 10 min of not wearing a face mask [36].

It has been reported that securing the upper part of the face mask with adhesive tape might help to improve NIBUT by limiting the flow of air that enters in contact directly to the eyes. A recent study measured NIBUT after 8 h of a working day in two subsequent days, the first day the participants were using the face mask without adhesive tape to secure the upper part of the face mask to the skin and on the second day, the participants were using face mask secured with adhesive tape to the skin [43]. A similar study found significant improvement in FBUT after 15 days of using a face mask secured with adhesive tape to the skin [8]. Although the practice of placing adhesive tape to secure the upper part of the face mask to the skin is a commonly carried out practice, it can have adverse effects to the skin. The mechanical irritation of the skin caused by the repeated application and removal of adhesive can result in skin damage, leading to a compromise in skin integrity. Such damage creates openings that allow the entry of foreign substances and potential pathogens, increasing the risk of infections [44]. In general, the use of adhesive tape to secure the upper part of the face mask to the face skin has generally been well tolerated, with few occasional adverse events such as skin erythema and irritative symptoms after prolonged use [11,18].

A cross-sectional study on healthcare workers compared the presence of signs and symptoms of dry eye between those using surgical face masks and N95 face mask. They found that both types of face masks had a significant reduction in FBUT, but the N95 face mask users showed significantly greater reductions in TBUT (*p* < 0.042) and in fluorescein corneal staining (*p* < 0.038) [10]. These findings suggest that different types of face masks have different severity on the effect at the ocular surface. A summary of selected studies evaluating mask-associated dry eye (MADE) syndrome is presented in Table 2.

These previous studies suggest that MADE is directly associated with the amount of time and extended wear of face mask use, and that there is a significant detrimental effect on TBUT. Some limitations of these previous studies are that most did not evaluate a control group, they did not take into consideration the expected increase in screen exposure time because of the pandemic, or also the fact that dry eye symptoms and signs increase as the day progresses. These results differ from the current study which did not find short term changes in NIBUT after immediate removal or placement of surgical face mask. In the present study, only the immediate effect of face mask use on NIBUT was evaluated, while the former studies found reductions in TBUT after 10 min, 6 h, 9 h, a complete day, 15 days, or 3 months of using face mask. This might mean that the deleterious effect of face masks on tear film stability is not immediate but dependent on their continuous use and extended wear time. 

Although it is frequent in the general population to wear spectacles or contact lenses, the information on the combined effect of face mask with contact lenses or spectacles is scarce. The impact of spectacles or contact lenses on the ocular surface has been studied, and it has been found that the use of spectacles does not generate a negative impact on the ocular surface or NIBUT [45]. On the contrary, using contact lenses has been found to generate a detrimental effect on the ocular surface, including a reduction in NIBUT [46]. The use of a face mask will also cause other visual and adaptive difficulties in those who wear spectacles, such as blurred vision, fogging, and discomfort in the ears [47]. A study reported that people who wear spectacles and face mask have more adaptative difficulties and a greater risk of not wearing the face mask, and those who have contact lenses may feel more comfortable with the use of the face mask, facilitating the appropriate usage of the face mask [47]. However, another study found that contact lens users can further increase dry eye symptoms and visual difficulties with the use of face masks [48].

The diagnosis of DED relies on several tests, usually involving a combination of (1) symptom questionnaires, like the OSDI or dry eye questionaries (DEQ5); (2) evaluation of tear film stability either with fluorescein TBUT or with NIBUT; and (3) ocular surface staining with fluorescein and lissamine green [12]. Currently, while the COVID-19 pandemic continues, ophthalmic evaluations are usually performed with patients wearing a face mask. This study evaluated the impact of wearing a face mask during NIBUT testing with K5M and found that there are no significant differences on NIBUT first values when it is measured while the patient wears either a face mask, a tape-secured face mask, or no face mask. According to this finding, tear film stability tests can be performed reliably in patients using a face mask, and the removal of the face mask or the placement of a tape to secure it do not have a significant short-term impact on NIBUT values. However, it was found a poor ICC in NIBUT measurements when using face mask and tape-secured face mask. Meaning there is increased variability in the measurements of NIBUT when using face mask or a tape-secured face mask. The ICC without face mask was moderate, therefore NIBUT may show less variability without face mask. In addition, the Bland–Altman agreement and the correlation between the measurements was limited.

This study has limitations. Only the short-term or immediate effect of face mask use was evaluated. Perhaps, as previous studies have shown, face masks need to be worn continuously for extended periods of time to cause changes in NIBUT, but further studies are needed to confirm this. An intrinsic limitation of NIBUT is its limited repeatability because of the high variability of the tear film [37]. Another limitation of the study is that the participants were only asked to self-report the ocular diseases or risk factors for ocular surface disease mentioned in exclusion criteria that could impact on the NIBUT evaluation, but did not had a comprehensive ocular evaluation that included signs of dry eye or other ocular pathologies. Only NIBUT was performed because the aim was only to assess the short-term impact of face mask use on tear film stability. It is important to note that, due to the pandemic, all participants in our study were using face masks for prolonged periods of time before their participation because current institutional regulations demand their continuous use while in the hospital facility. It was not possible to design a study with a control session in which the participants were without a face mask for a prolonged period because of said regulations. This study only evaluated one type and design of surgical face mask, and there might be differences between types and models of surgical face masks. The strengths of this study are that a well-designed methodology was performed, in which all participants had the same four evaluations with different order and day of measurement. This ensured that the most precision possible out of the objective K5M measurements was obtained.

In conclusion, this pilot study found no significant short-term effect of surgical face mask use on NIBUT. There is a need for further studies with larger sample sizes, more diverse population including healthy, dry eye, and control groups to extrapolate the finding of this pilot study to a wider population and to analyze the short-term and long-term effects of face masks on TBUT, ocular surface and their relationship with dry eye symptoms associated with face mask use.

## Figures and Tables

**Table 1:** Non-invasive break-up time (NIBUT) without a face mask, with a face mask, and with a tape-secured face mask.

	NIBUT Mean ± SD (Seconds)
No face mask	8.9 ± 3.7
Face mask	10.2 ± 4.1
Tape-secured face mask	8.4 ± 3.8
	**Mean Dif. (95% CI); *p***
No face mask vs. face mask	−1.2 (−3.4–0.9); *p* = 0.24
No face mask vs. tape-secured face mask	0.5 (−1.5–2.6); *p* = 0.91
Face mask vs. tape-secured face mask	−1.8 (−4.0–0.3); *p* = 0.11

**Table 2:** Summary of selected studies evaluating mask-associated dry eye (MADE) syndrome.

Author. [Reference]	Year, Country	N=, Population	Evaluation	Key Comments and Findings
Moshirfar M. et al. [3]	2020, USA	0	None	Face mask could produce ocular irritation and dryness
Boccardo L. et al. [4]	2022, Italy	3605, general population	Survey	18.3% experienced MADE. Those with previous DED symptoms were more likely to have worsening of DED symptoms.
Chadwick O. et al. [5]	2020, UK	1	Comment/ Case report	Ill-fitting face masks can cause exposure keratopathy in the setting of an anesthetized cornea
Giannaccare G. et al. [7]	2020, Italy	107, medical students	Survey/Letter to the editor	24.3% used VDT in the last month for 6 h/day or more. 67.3% wore face mask > 6 h/day. 10.3% described appearance or worsening of ocular discomfort symptoms, 19.6% reported the need for daily use of tear substitutes. OSDI scored ≥ 15 (pathological) was 21, 57%. Mixed causes for DED were observed: DES and face mask use.
Pandey S. et al. [5]	2021, India	0	Letter to the editor	Important to use face mask properly. Mixed component for the increase on DED in the pandemic, DES and face mask use.
Scalinci S. et al. [28]	2021, Italy	67, previous DED	Survey: OSDI	Retrospective comparison of OSDI scores in spring 2019 (pre pandemic) vs. spring 2020 (pandemic). Heavy face mask use (≥6 h/day, and ≥5 day/week, in the last 2 months, is associated with an increase in OSDI scores.
Krolo I. et al. [29]	2021, Croatia	203, patients attending eye clinic	Survey: Modified OSDI	Surgical face mask use 3–6 h/day had higher OSDI compared to <3 h/day use:15.3 (IQR = 8.3–47.7) vs. 8.3 (IQR = 0.0–35.1); *p* = 0.001). Prior DED had greater worsening of OSDI during face mask use (54.8% vs. 17.7%; *p* < 0.001). Approximately half experienced worsening of DED symptoms while wearing face mask.
Aksoy M. et al. [31]	2021, Turkey	52, outpatient	FBUT, OSDI, DED tests.	3-layered face mask users >8 h/day had significant reduction in FBUT compared with the beginning of the day before face mask use. Taping down the mask improved FBUT. No control group.
Mastropasqua L. et al. [30]	2021, Italy	62 DED, 62 non-DED healthy	FBUT, DEQS, DED tests	The greater the use of surgical face mask, the more FBUT, symptoms and other DED parameters were reduced in healthy and DED patients. After 3 months of face mask use, only those using >6 h/day had significant FBUT reductions. No control group.
Nair S. et al. [36]	2022, India	50, Healthy workers	NIBUT, OSDI, other tests	N95 face mask. Taping of the upper face mask edge results in significantly better ocular surface stability and decrease in DED symptoms
Arriola-Villalobos P. et al. [33]	2021, Spain	31, DED	TBUT, and other tests	Removal of face mask for 10 min improves tear film stability in patients with moderate-to-severe DED.
Azzam S. et al. [34]	2022, Israel	30 surgical mask, 30 n95. Health-care workers	TBU, DED signs and symptoms	Cross-sectional study. Both types of face masks had reduced FBUT, but the N95 face mask users had greater reductions on TBUT (*p* < 0.042) and fluorescein staining (*p* < 0.038).
Esen Baris M. et al. [32]	2022, Turkey	66 eyes of 33 subjects	TBUT and other signs	Significant reduction in FBUT after 9 h of face mask use when compared to basal evaluation with face mask at the start of the day.
This study. Mohamed-Noriega. et al.	2023, Mexico	26 eyes, 26 health-care workers	NIBUT	No significant short-term differences were observed on NIBUT between using surgical face mask, after removal of face mask or after securing face mask with adhesive tape

DED: dry eye disease, VDT: video terminal displays, OSDI: Oscular surface disease index questionary, DES: digital eye strain, FBUT: fluoresceine tear break-up time, NIBUT: Non-invasive tear break-up time, DEQS: Dry Eye-related Quality of life Score.

## Data Availability

The data used to support the findings of this study are included within the article. All the information regarding this project, files, worksheets, spreadsheets, and databases are readily available upon written request to the corresponding author.

## Supplementary Materials

The following supporting information can be downloaded at: https://www.mdpi.com/article/10.3390/jcm12247727/s1, Figure S1: Bland–Altman plot of NIBUT measurements without a face mask. (A): In the 1st and 3rd sessions the average difference was −0.52, suggesting a small average difference between one measurement versus the other. (B): In the second and fourth sessions the average difference was 0.77, suggesting a small average difference between one measurement versus the other; Figure S2: Scatter-plot analysis of NIBUT values of the four measuring sessions (A–D). The x-axis in all diagrams corresponds to the first measurement in each session, and the y-axis to the second measurement.
