# Supplementary material for: Face Mask and Tear Film Stability: A Pilot Study of the Objective Measurement of Tear Break-Up Time
Source: J Clin Med. 2023 Dec 16;12(24):7727. doi: 10.3390/jcm12247727 (PMC10743798; doi:10.3390/jcm12247727)
Supplement: Supplementary file 1 [file jcm-12-07727-s001.zip › Supplemental Figure S1.pdf]

Supplemental Material

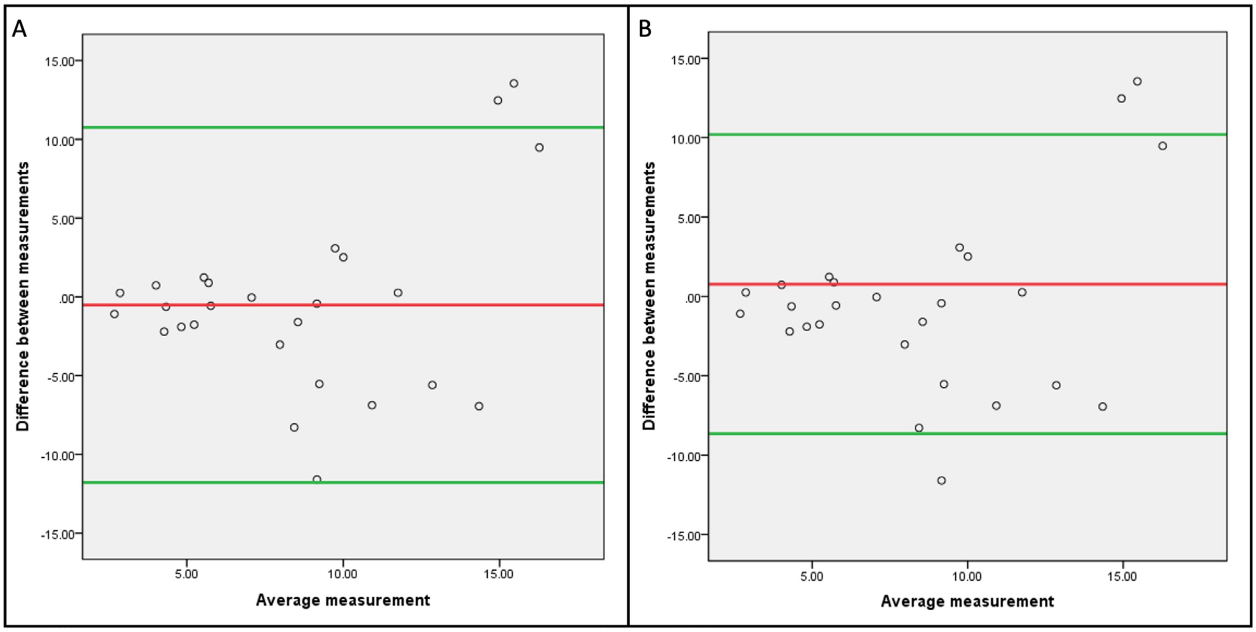

Supplemental Figure S1. Bland-Altman plot of NIBUT measurements without a face mask. **A: In the 1st and 3rd sessions the average difference was -0.5**2, suggesting a small average difference between one measurement versus the other. **B**: In the 2nd and 4th sessions the average difference was 0.77, suggesting a small average difference between one measurement versus the other.
