# Supplementary material for: Face Mask and Tear Film Stability: A Pilot Study of the Objective Measurement of Tear Break-Up Time
Source: J Clin Med. 2023 Dec 16;12(24):7727. doi: 10.3390/jcm12247727 (PMC10743798; doi:10.3390/jcm12247727)
Supplement: Supplementary file 1 [file jcm-12-07727-s001.zip › Supplemental Figure S2.pdf]

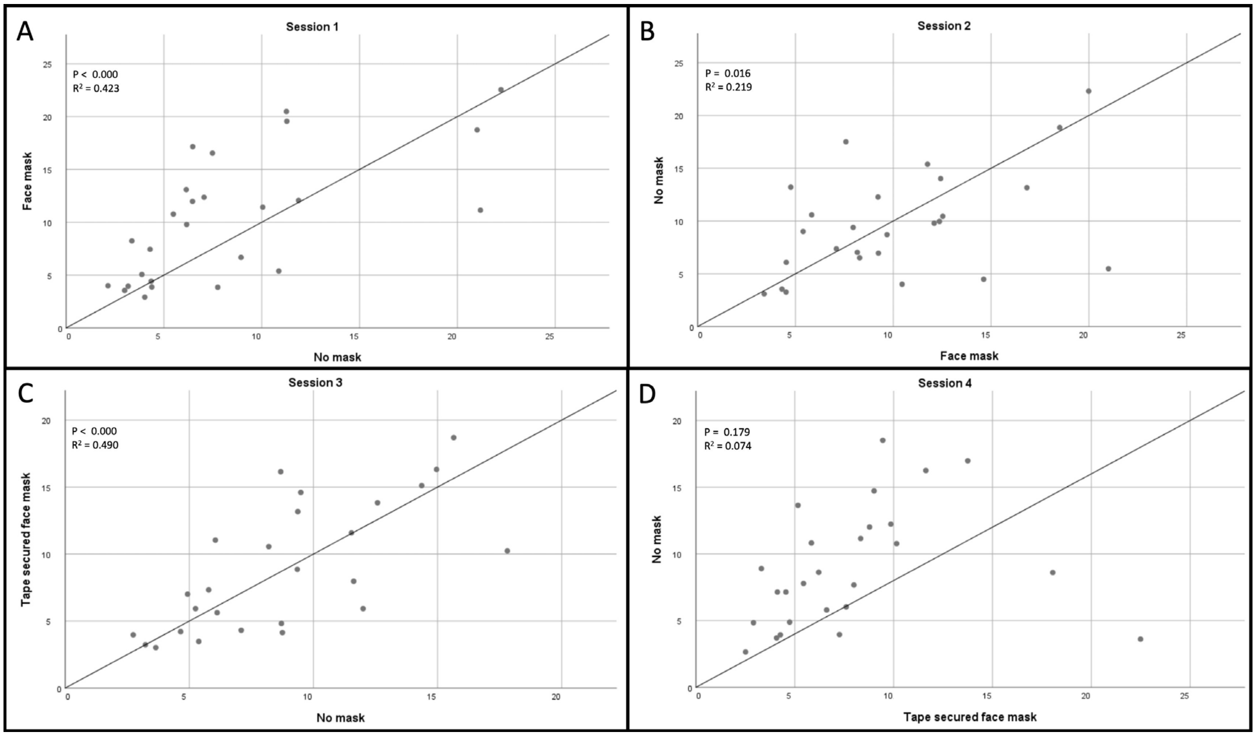

Supplemental Figure S 2. Scatter-plot analysis of NIBUT values of the four measuring sessions (A-D). The x-axis in all diagrams corresponds to the first measurement in each session, and the y-axis to the second measurement.
